# Association between Dietary Habits and Pancreatitis among Individuals of European Ancestry: A Two-Sample Mendelian Randomization Study

**DOI:** 10.3390/nu15051153

**Published:** 2023-02-24

**Authors:** Xiaotong Mao, Chunyou Huang, Yuanchen Wang, Shenghan Mao, Zhaoshen Li, Wenbin Zou, Zhuan Liao

**Affiliations:** 1 Department of Gastroenterology, Changhai Hospital, Navy Medical University, Shanghai 200433, China; 2 Shanghai Institute of Pancreatic Diseases, Shanghai 200433, China

**Keywords:** pancreatitis, food intakes, Mendelian randomization, lifestyle

## Abstract

Dietary factors are believed to potentially influence the risk of pancreatitis. Here, we systematically investigated the causal relationships between dietary habits and pancreatitis by using two-sample Mendelian randomization (MR). Large-scale genome-wide association study (GWAS) summary statistics for dietary habits were obtained from the UK Biobank. GWAS data for acute pancreatitis (AP), chronic pancreatitis (CP), alcohol-induced AP (AAP) and alcohol-induced CP (ACP) were from the FinnGen consortium. We performed univariable and multivariable MR analyses to evaluate the causal association between dietary habits and pancreatitis. Genetically driven alcohol drinking was associated with increased odds of AP, CP, AAP and ACP (all with *p* < 0.05). Genetic predisposition to higher dried fruit intake was associated with reduced risk of AP (OR = 0.280, *p* = 1.909 × 10^−5^) and CP (OR = 0.361, *p* = 0.009), while genetic predisposition to fresh fruit intake was associated with reduced risk of AP (OR = 0.448, *p* = 0.034) and ACP (OR = 0.262, *p* = 0.045). Genetically predicted higher consumption of pork (OR = 5.618, *p* = 0.022) or processed meat (OR = 2.771, *p* = 0.007) had a significant causal association with AP, and genetically predicted higher processed meat intake increased the risk of CP (OR = 2.463, *p* = 0.043). Our MR study showed that fruit intake may be protective against pancreatitis, whereas dietary intake of processed meat has potential adverse impacts. These findings may inform prevention strategies and interventions directed toward dietary habits and pancreatitis.

## 1. Introduction

Pancreatitis is a complex, progressive and destructive inflammatory disease of the pancreas with a high risk of morbidity and mortality. Acute pancreatitis (AP) has an estimated global incidence of 33.74 cases and 1.16 deaths per 100,000 person–years and ranks among the most common gastrointestinal cause of hospital admissions [1,2]. Approximately 20% of patients with a first episode of AP develop recurrent acute pancreatitis (RAP), and 3–35% of patients will progress to chronic pancreatitis (CP) over 3–8 years [3,4,5]. CP is a serious condition that significantly deteriorates patients’ quality of life and decreases life expectancy, complications of which include pancreatic exocrine insufficiency, diabetes mellitus and pancreatic cancer.

Cholelithiasis, alcoholism, smoking and hyperlipidemia are common causes of pancreatitis, often in combination with other risk factors, including genetic factors or anatomic variants. Adding to these known risk factors, the role of dietary habits has received increasing attention as a potential risk factor for pancreatitis [6]. To date, several studies have been published to investigate the association between dietary factors and incidence of AP. A series of population-based prospective cohort studies conducted by Oskarsson et al. suggested associations between the incidence of non-gallstone-related AP and the consumption of vegetables, fish and high-glycemic load foods [7,8,9]. Another multiethnic cohort study showed that the dietary intake of food rich in saturated fat and cholesterol was associated with an increased risk of gallstone-related AP, whereas fiber intake protected against AP related and unrelated to gallstones [10]. Additionally, the association between vitamins and pancreatitis has received growing attention [11]. It has been a challenge to establish a link between diet and pancreatitis, and case-control studies are prone to recall bias. Currently, the relationship between food intake and the risk of pancreatitis, especially for CP, has not been fully elucidated yet.

Mendelian randomization (MR) is a popular approach that uses the unique properties of genotype to investigate causal associations between exposures and outcomes [12]. It uses measured genetic variants robustly related to an exposure of interest as instrumental variables (IVs), and these variants are randomly allocated across the population at meiosis and conception, mimicking a randomized controlled setting. The MR design can avoid the effects of the potential residual confounders and overcome the reverse causation bias [13]. To date, several studies using the Mendelian randomization to estimate the causal effects of multiple potential exposures on pancreatitis have been reported. Hansen et al.’s study revealed that genetic variants associated with increased plasma levels of triglycerides increase the risk of AP [14]. Yuan et al. investigate the causal associations of gallstone disease, diabetes, serum calcium, triglycerides, smoking and alcohol in AP and CP [15]. More recently, Mi et al. reported that genetically elevated triglyceride levels and reduced degree of unsaturation in fatty acids were associated with the increased risk of pancreatitis [16]. These studies have provided new insights toward novel strategies for the prevention and treatment of pancreatitis.

Considering there was a lack of evidence pertaining to the relationship between dietary habits and pancreatitis, we used a two-sample MR approach to explore the causal effects of eighteen genetically proxied food intake patterns on the risks of AP, CP, alcohol-induced AP (AAP) and alcohol-induced CP (ACP), using publicly available summary statistics from genome-wide association studies (GWAS). Our study may elucidate the potential genetic mechanisms between dietary habits and pancreatitis and provide scientific evidence for disease primary prevention.

## 2. Materials and Methods

### 2.1. Study Design

**Figure 1:**
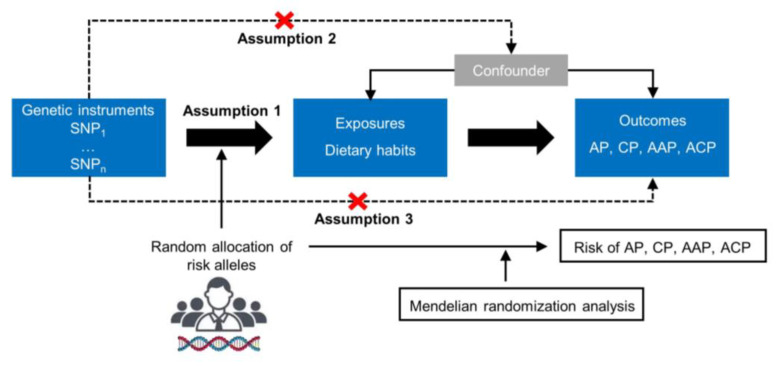
Study design overview and assumptions of the Mendelian randomization design. The dashed lines indicate possible causal effects between variables that may be against the Mendelian randomization assumptions. SNPs: single nucleotide polymorphisms. AP: acute pancreatitis. CP: chronic pancreatitis. AAP: alcohol-induced acute pancreatitis. ACP: alcohol-induced chronic pancreatitis.

A two-sample MR design was utilized to investigate the causal effect of dietary habits on different types of pancreatitis (Figure 1). Single nucleotide polymorphisms (SNPs) associated with these risk factors were selected as IVs. The MR design is based on three core assumptions: (1) genetic IVs must be closely related to the exposure; (2) the IVs are irrelevant to various confounders; (3) the selected IVs influence the outcome only via exposure. The datasets used in our study are retrieved from public databases and received ethical approval prior to implementation. This study, therefore, did not require additional ethical approval.

### 2.2. GWAS Summary-Level Data of Dietary Habits and Pancreatitis

The GWAS summary statistics of alcohol drinking were obtained from the GWAS and Sequencing Consortium of Alcohol and Nicotine use (GSCAN) [17]. We acquired GWAS summary data of 17 dietary patterns from the UK Biobank, which is a large prospective cohort including approximately 500,000 participants with genetic and various phenotypic information [18]. GWAS summary data for pancreatitis were obtained from the FinnGen consortium. The R7 release (June 2022) of the FinnGen consortium data was used (https://r7.finngen.fi/, accessed on 28 November 2022), which contains 4648 cases and 273, 442 controls for AP, 2659 cases and 273,442 controls for CP, 705 cases and 308,449 controls for AAP and 1425 cases and 307,729 controls for ACP.

### 2.3. Genetic Instrument Selection

To explore the causal association between genetically predicated dietary habits and pancreatitis, SNPs were used as IVs. We selected eligible genetic IVs from European-descent GWAS summary datasets and followed a series of quality control procedures. The SNPs highly related with each exposure (*p* < 5 × 10^8^) were extracted. Second, we performed Linkage disequilibrium (LD)-based clumping procedure with *r*^2^ < 0.01 and a window size of 10,000 kb to ensure that each IV was independent. LD was estimated using the 1000 Genomes EUR reference panel. Third, the *F* statistic was used to assess the genetic instrument strength and avoid bias caused by weak IVs. The *F* statistic is a measure of instrument strength [19]. We evaluated the power of each single IV using the *F* statistics (*F* = beta^2^/se^2^). A general *F* statistic for each dietary habit was also calculated using the following equation:

$$F=\frac{\left(n-k-1\right)R^{2}}{k\left(1-R^{2}\right)}$$

where *n* is the sample size of the exposure dataset, *k* is the number of SNPs and *R*^2^ is the portion of exposure variance explained by the genetics. We calculated the *R*^2^ using the following formula [20]:

$$R^{2}=\frac{2\times \mathrm{EAF}\times \left(1-\mathrm{EAF}\right)\times {\mathrm{beta}}^{2}}{2\times \mathrm{EAF}\times \left(1-\mathrm{EAF}\right)\times {\mathrm{beta}}^{2}+2\times \mathrm{EAF}\times \left(1-\mathrm{EAF}\right)\times n\times {\mathrm{se}}^{2}}$$

where EAF is the effect allele frequency, beta is the estimated genetic effect and se is the standard error of the genetic effect.

An *F* statistic greater than 10 was considered a strong genetic variant [19]. In this study, all *F* statistics were higher than 10, indicating little chance of weak-instrument bias based on the summary statistics.

### 2.4. Univariate and Multivariate MR Analysis

The random-effect inverse-variance weighted (IVW) method was used as the primary methodology for the main analysis of MR. In addition, we used three different methods (weighted median, MR Egger and the MR-PRESSO-corrected approach) to enable valid estimation in the presence of horizontal pleiotropy. Horizontal pleiotropy occurs when the selected IV affects other traits outside of the pathway of the candidate exposure and has an impact on the target outcome or when the IV has a direct effect on the target outcome [21]. Violation of the ‘no horizontal pleiotropy’ assumption can lead to severe bias in MR. The weighted median method combines data on multiple genetic variants into a single causal estimate and provides unbiased causal effects if at least half of the chosen SNPs are valid [22]. The MR-Egger method does not force the regression line through the origin, allowing the included IVs to demonstrate unbalanced pleiotropy [23]. The MR-PRESSO approach was used to detect horizontal pleiotropic outliers, and causal effects were further analyzed with the IVW method after excluding the outliers [24]. The MR-PRESSO method was used to detect the existence of pleiotropy. Moreover, selected IVs are sometimes associated with multiple aspects of exposures. Such heterogeneity could undermine the ability to infer causality for particular dimensions of heterogeneous exposures [25]. Thus, the Cochran’s Q test were employed to evaluate the heterogeneity among IVs. To clarify whether significant causal dietary habits were directly associated with the risk of pancreatitis rather than being mediated by hub exposures, multivariable analysis was performed to adjust for known confounders.

### 2.5. Statistical Analyses

The univariable and multivariable MR analysis was performed using R software (R version 4.2.1, R Foundation for Statistical Computing, Vienna, Austria) with the R packages “TwoSampleMR” (https://github.com/MRCIEU/TwoSampleMR accessed on 28 November 2022), and the MR-PRESSO was conducted using the R package “MR-PRESSO” (https://github.com/rondolab/MR-PRESSO, accessed on 28 November 2022). The data visualization was performed using R package “forestploter”. The results are reported as odds ratios (OR) with corresponding 95% confidence intervals (CIs). Two-sided *p*-values < 0.05 were considered statistically significant.

## 3. Results

### 3.1. Genetic Instruments for Eighteen Dietary Habits

The detailed information of each participating GWAS study are shown in Table 1. Overall, eighteen kinds of dietary patterns were included in the analyses. The number of SNPs for each dietary habit ranged from 9 to 124. Detailed information of IVs for eighteen dietary habits was listed in Supplementary Table S1. Across the dietary exposures that were examined, the F statistics of the obtained SNPs were all greater than the empirical threshold of 10, suggesting that the results are less likely to deviate owing to the influence of weak IVs.

### 3.2. Causal Effects of Dietary Habits on AP and CP

**Figure 2:**
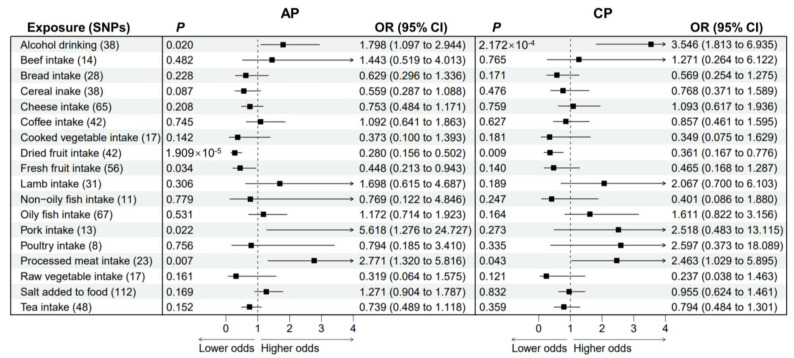
Forest plot to visualize the causal effect of dietary habits on AP and CP using the inverse variance-weighted method. AP: acute pancreatitis. CP: chronic pancreatitis. SNPs: single nucleotide polymorphisms. OR: odds ratio. CI: confidence interval.

In the primary univariable MR analyses, four causal associations from eighteen dietary habits to AP were identified (Figure 2; Supplementary Table S2), while two causal associations were observed for CP (Figure 2; Supplementary Table S3). Genetically driven alcohol drinking increased the risk of AP (OR = 1.798; 95% CI, 1.097–2.944; *p* = 0.020) and CP (OR = 3.546; 95% CI, 1.813–6.935; *p* = 2.172 × 10^−4^). Genetically predicted dried fruit intake were strongly associated with a reduced risk of both AP (OR = 0.280; 95% CI, 0.156–0.502; *p* = 1.909 × 10^−5^) and CP (OR = 0.361; 95% CI, 0.167–0.776; *p* = 0.009). We also found evidence that genetic predisposition to increased consumption of fresh fruit was protective against AP (OR = 0.448; 95% CI, 0.213–0.943; *p* = 0.034). On the contrary, genetically predicted processed meat intake levels were significantly associated with the risk of both AP (OR = 2.771; 95% CI, 1.320–5.816; *p* = 0.007) and CP (OR = 2.463; 95% CI, 1.029–5.895; *p* = 0.043). Genetic liability to pork intake was associated with a higher risk of AP (OR = 5.618; 95% CI, 1.276–24.727; *p* = 0.022) but was not associated with CP (OR = 2.518; 95% CI, 0.483–13.115; *p* = 0.273). There was no evidence for potential heterogeneity or pleiotropy biasing our findings based on the Cochran’s Q test and MR-PRESSO global test (all *p* values > 0.05; Supplementary Tables S2 and S3).

### 3.3. Causal Effects of Dietary Habits on AAP and ACP

**Figure 3:**
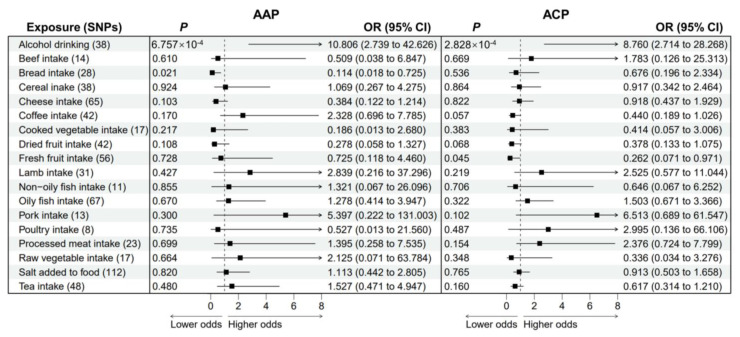
Forest plot to visualize the causal effect of dietary habits on AAP and ACP using the inverse variance-weighted method. AAP: alcohol-induced acute pancreatitis. ACP: alcohol-induced chronic pancreatitis. SNPs: single nucleotide polymorphisms. OR: odds ratio. CI: confidence interval.

Subsequently, we studied the causal associations between dietary habits and alcohol-induced pancreatitis (Figure 3; Supplementary Tables S4 and S5). Genetic liability to alcohol drinking was strongly associated with higher odds of AAP (OR = 10.806; 95% CI, 2.739–42.626; *p* = 6.757 × 10^−4^) and ACP (OR = 8.760; 95% CI, 2.714–28.268; *p* = 2.828 × 10^−4^). When we analyzed the association between genetically predicted alcohol drinking and ACP, we observed possible pleiotropy (*P*_pleiotropy_ = 0.006) and heterogeneity (*P*_heterogeneity_ = 0.004) (Supplementary Table S5). Thus, we performed a MR-PRESSO analysis to detect potentially pleiotropic outliers. After removing outlier SNPs, the relationship remained stable in the MR-PRESSO-corrected results (OR = 9.884; 95% CI, 3.659–26.698; *p* = 6.213 × 10^−6^). Genetic predisposition to bread intake was significantly associated with a reduced risk of AAP (OR = 0.114; 95% CI, 0.018–0.725; *p* = 0.021), while genetically predicted fresh fruit intake was associated with lower odds of ACP (OR = 0.262; 95% CI, 0.071–0.971; *p* = 0.045). There was possible pleiotropy for bread intake (*P*_heterogeneity_ = 0.042; Supplementary Table S4). After removing potential outlier SNPs, the negative association between bread intake and AAP remained significant (OR = 0.148; 95% CI, 0.026–0.861; *p* = 0.033). Genetic predisposition to higher coffee intake levels could be a protective factor against ACP (OR = 0.440; *p* = 0.057). It should be noted that genetic liability to dried fruit intake trended toward a decreased risk of AAP (OR = 0.278; *p* = 0.108) or ACP (OR = 0.378; *p* = 0.068) but did not achieve statistical significance.

### 3.4. Multivariable MR Analysis of Pancreatitis

**Figure 4:**
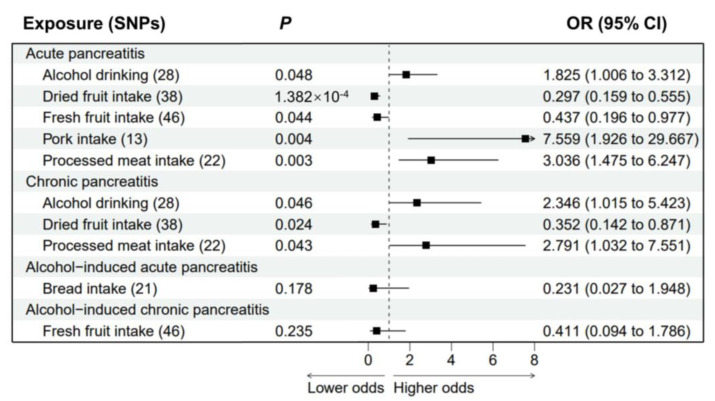
The association between adjusted dietary habits and pancreatitis by multivariable Mendelian randomization. Associations between dietary habits and AP or CP were adjusted for cholelithiasis (FinnGen consortium). Association between dietary habits and AAP or ACP were adjusted for alcohol drinking (GWAS and Sequencing Consortium of Alcohol and Nicotine use). SNPs: single nucleotide polymorphisms. OR: odds ratio. CI: confidence interval.

Considering that gallstone disease is common among pancreatitis patients, we performed multivariable MR analyses to assess the association between dietary habits and AP or CP after adjusting for cholelithiasis (Figure 4). The association between genetic predisposition to alcohol drinking (adjusted OR = 1.825; *p* = 0.048), dried fruit intake (adjusted OR = 0.297; *p* = 1.382 × 10^−4^), fresh fruit intake (adjusted OR = 0.437; *p* = 0.044), pork intake (adjusted OR = 7.559; *p* = 0.004) or processed meat intake (adjusted OR = 3.036; *p* = 0.003) and AP remained significant in multivariable MR models. No significant association remained between pork intake and AP (*p* = 0.055; Supplementary Table S6) after adjusting for genetically predicted body mass index (BMI), suggesting that this association could be affected by BMI. Genetic predisposition to alcohol drinking (adjusted OR = 2.346; *p* = 0.046), dried fruit intake (adjusted OR = 0.352; *p* = 0.024) or processed meat intake (adjusted OR = 2.791; *p* = 0.043) also had similar significant causal effects on CP after adjusting for the genetic risk of cholelithiasis, which confirmed the robustness of the results. Finally, we performed multivariable analyses on alcohol-induced pancreatitis by adjusting for alcohol drinking (Figure 4). The protective effects of genetic-predicted bread intake (*p* = 0.178) and fresh fruit intake (*p* = 0.235) were no longer statistically significant.

## 4. Discussion

The human pancreas is a composite organ that serves two important functions: the production of enzymes for the digestion of food (exocrine function) and the secretion of hormones to regulate glucose metabolism (endocrine function). On the one hand, the pancreas releases three main groups of digestive enzymes, including amylase, trypsin and lipase, which can, respectively, digest carbohydrates, proteins and digest fats into their basic components so that they can be absorbed and utilized by the body [26]. On the other hand, animal studies demonstrated that dietary constituents affected the development of pancreatic functions and the secretion pattern of digestive enzymes [27,28,29]. It is generally accepted that the key event for the initiation of pancreatitis involves pathologic autodigestion triggered by prematurely activated pancreatic enzymes within the pancreas [30]. Additionally, unreasonable dietary habits may result in metabolic changes or disturbances, leading to the development of pancreatitis. However, there has been limited data about the influence of dietary habits on the risk of pancreatitis so far. Here, we illustrate the relationship between dietary habits and pancreatitis using MR analysis and large-scale GWAS data and identify specific food intake that might be causally associated with pancreatitis risk.

Alcohol drinking is a well-known lifestyle risk factor for both AP and CP, which accounts for 20% of the aetiologies in AP patients and 40–70% of the aetiologies in CP patients [2,5]. Moreover, excessive alcohol consumption is an important risk factor for the recurrence of AP, as well as for progression to CP [2]. Our MR results confirmed the causal associations between genetically predicted alcohol drinking and all four types of pancreatitis. In line with the expectation, the odds ratio for alcohol use in AAP or ACP was higher than the odds ratio in AP or CP. The effects of alcohol drinking on AP and CP were partially attenuated after adjusting for cholelithiasis, suggesting that this association could be influenced by gallstone disease. The previous MR study by Yuan et al. did not support the positive association between alcohol consumption and AP [15]. It is possible that the limited sample size (1762 cases and 121,348 controls) might have affected the statistical power to identify significant associations in their results. In this present study, we take advantage of the latest available data for pancreatitis in the FinnGen consortium, which included a substantially higher number of cases and controls (4648 cases and 273, 442 controls).

We found that genetically predicted consumption of fruit (both fresh and dried) was inversely associated with the risk of AP, and genetic predisposition to dried fruit intake was suggestively protective of CP. Our results are consistent with findings by Setiawan et al., which showed that fruit intake was associated with a reduced risk of AP [10]. Notably, high intake of fruits has been shown to decrease the risks of gallstone diseases in previous studies [31], and the association between fruit intake and pancreatitis was no longer significant in non-gallstone-related AP cohorts [7,10]. Considering that gallstone disease is common among patients with pancreatitis, we performed multivariable MR analyses to adjust for the genetic risk of cholelithiasis. Inspiringly, the inverse association for genetically predicted higher fruit intake levels remained prominent in multivariable MR models, suggesting that fruit consumption might be the independent protective factor against AP and CP. The high content of antioxidants in fruits may prevent the onset of pancreatitis through reduction of the basal oxidative stress level. Moreover, dietary fibers from fruits have been reported to be protective against the occurrence of AP [32]. Genetic liability to fresh fruit intake significantly decreased the risk of ACP; however, the association did not persist after adjusting for cholelithiasis, suggesting that this association is not robust enough.

Setiawan et al. reported that diets rich in saturated fat and cholesterol, including red meat and eggs, were positively linked with a higher risk of gallstone-related AP [10]. Another cross-sectional study from China showed high meat consumption was associated with AP risk; however, the association was not significant after adjustment of confounding factors [33]. Our results provided evidence supporting the positive association between genetically predicted processed meat intakes and pancreatitis risk. Additionally, genetic liability to higher pork intake levels significantly increased the risk of AP. Long-term high-fat diet exposure and gallstones may work synergistically to promote the occurrence of AP [34], and high-fat and cholesterol diets have been reported as risk factors for gallstone in previous studies [35,36]. After adjustment for cholelithiasis, the positive relationship between processed meat intake and pork intake remained significant, suggesting that consumption of processed meat and pork may also affect the risk of pancreatitis via other mechanisms. We did not observe a positive association between genetically predicted beef intake and pancreatitis, which is consistent with a previous prospective case-control study [37].

Two Swedish population-based prospective studies by Oskarsson et al. showed a significant inverse association between consumption of vegetable or fish and the risk of non-gallstone-related AP [7,9]. We observed that genetic predisposition to the intake of cooked vegetables or raw vegetables appeared to decrease risks of both AP and CP, but this association did not reach statistical significance. We did not find evidence to support an association between fish consumption and pancreatitis. As proposed by Setiawan et al., the protective effects of fish intake against AP are likely to be ethnic-specific [10]. Currently, it is still controversial whether coffee drinking decreases risk of pancreatitis. Two studies have drawn differing conclusions regarding the relationship between coffee drinking and non-gallstone related AP [10,38]. The earlier cohort study in the United States showed coffee drinking is associated with reduced risk of alcohol-associated pancreatitis [39]. Our MR results did not support a significant association between coffee consumption and AP, CP or AAP. Genetically predicted higher coffee intake levels tended to reduce the risk of ACP, although only borderline statistically significant. Notably, we observed an inverse association of genetically predicted bread intake with AAP, whereas this relationship was no longer statistically significant after adjusting for alcohol consumption.

Our study has several significant strengths. First, the MR design is suitable for causal inference. As an alternative to randomized controlled trials, the MR design is less vulnerable to bias from reverse causation and unmeasured confounding, which are prevalent in conventional observational studies. Second, the present study systematically analyzed the relationship between pancreatitis and a wide range of dietary habits, some of which have never been reported in previous studies. The large sample size and the usage of strong instruments (all SNPs had *F* statistics > 10) guaranteed enough statistical power. Third, population stratification bias was minimized because all GWAS summary statistic data analyzed in this study were generated from individuals of European descent. Fourth, GWAS data for pancreatitis in this study were obtained from the FinnGen consortium, while data for food intakes were from the UK Biobank. The design avoided population overlap between exposures and outcomes, thereby decreasing the likelihood of type 1 error rate due to weak instrument bias [40].

Nevertheless, some limitations in this MR study should be noted. First, MR analyses can be potentially biased by pleiotropic effects. As with all MR studies, pleiotropy in the MR setting was challenging. In this present study, we conducted various sensitivity analyses under different assumptions about the underlying nature of pleiotropy, most of which showed stable results. We also used MR-Egger intercept tests and MR-PRESSO analyses to detect widespread horizontal pleiotropy [23,24]. After removing potential outlier SNPs, we observed robust MR-PRESSO-corrected results. Second, all the participants included in this study were of European descent, this may limit the generalizability of our findings to other populations. Further studies are required to verify our findings in individuals of non-European descent. Third, the strength of evidence in MR studies depends considerably on the plausibility of the instrumental variable assumptions for the genetic variants. Canalization or developmental compensation buffers against the effect of the genetic variation, which could make it difficult to evaluate the gene–disease association. Thus, randomized controlled trials remain the gold standard for estimating the direct causal effect of interventions on health outcomes.

## 5. Conclusions

In summary, we systematically evaluated the potential causal relationship between dietary habits and pancreatitis. This MR analysis showed that genetically predicted dried fruit intake is causally associated with a reduced risk of AP and CP, while fresh fruit intake has potential preventive value against AP. In addition, processed meat intake was found to increase the risk of AP and CP, and pork intake was associated with AP risk. Our study contributes to a more targeted prevention strategy for pancreatitis by providing a better understanding of the possible roles of dietary patterns in the development of pancreatitis.

## Figures and Tables

**Table 1:** Summary of eighteen dietary habits for pancreatitis.

Exposures	SNPs (Number)	Unit	Sample (*n*)	Ancestry	*R*^2^ (%)	*F*	Consortium
Alcohol drinking	39	SD	335,394	European	0.54	46.69	GSCAN
Beef intake	17	SD	461,053	European	0.15	40.74	UK biobank
Bread intake	34	SD	452,236	European	0.31	41.36	UK biobank
Cereal intake	44	SD	441,640	European	0.45	45.37	UK biobank
Cheese intake	73	SD	451,486	European	0.62	38.58	UK biobank
Coffee intake	44	SD	428,860	European	0.73	71.67	UK biobank
Cooked vegetable intake	17	SD	448,651	European	0.14	37.00	UK biobank
Dried fruit intake	46	SD	421,764	European	0.45	41.44	UK biobank
Fresh fruit intake	58	SD	446,462	European	0.59	45.68	UK biobank
Lamb intake	33	SD	460,006	European	0.29	40.54	UK biobank
Non-oily fish intake	12	SD	460,880	European	0.11	42.29	UK biobank
Oily fish intake	73	SD	460,443	European	0.69	43.82	UK biobank
Pork intake	14	SD	460,162	European	0.12	39.49	UK biobank
Poultry intake	9	SD	461,900	European	0.06	30.81	UK biobank
Processed meat intake	24	SD	461,981	European	0.20	38.57	UK biobank
Raw vegetable intake	21	SD	435,435	European	0.18	37.39	UK biobank
Salt added to food	124	SD	462,630	European	1.30	49.13	UK biobank
Tea intake	50	SD	447,485	European	0.63	56.73	UK biobank

SNPs: single nucleotide polymorphisms. *R*^2^: phenotype variance explained by genetics. *F*: F statistics. SD: standard deviation. GSCAN: GWAS and Sequencing Consortium of Alcohol and Nicotine use.

## Data Availability

The GWAS summary data of AP, CP, AAP and ACP were downloaded from the FinnGen study (https://r7.finngen.fi/, accessed on 28 November 2022). All genome-wide association studies of dietary habits can be accessed via Open GWAS database (https://gwas.mrcieu.ac.uk/, accessed on 28 November 2022).

## Supplementary Materials

The following supporting information can be downloaded at: https://www.mdpi.com/article/10.3390/nu15051153/s1, Table S1: Genome-wide significant and independent SNPs that were used as instruments for eighteen dietary habits; Table S2: Results of the MR study testing causal association between dietary habits and AP; Table S3: Results of the MR study testing causal association between dietary habits and CP; Table S4: Results of the MR study testing causal association between dietary habits and AAP; Table S5: Results of the MR study testing causal association between risk factors and ACP; Table S6: Effect estimates for the association between dietary habits and pancreatitis in multivariable MR analysis.
